# Investigation of the relationship between ergocristinine and vascular receptors

**DOI:** 10.1016/j.toxrep.2023.05.005

**Published:** 2023-05-14

**Authors:** Jensen E. Cherewyk, Barry R. Blakley, Ahmad N. Al-Dissi

**Affiliations:** a Department of Veterinary Biomedical Sciences, Western College of Veterinary Medicine, University of Saskatchewan, Saskatoon, SK, S7N 5B4 Canada; b Department of Veterinary Pathology, Western College of Veterinary Medicine, University of Saskatchewan, Saskatoon, SK, S7N 5B4 Canada

**Keywords:** Ergot alkaloid, Ergocristine, Mycotoxin, Affinity, Molecular interactions, Molecular docking

## Abstract

Ergot alkaloids are secondary metabolites that exist in two configurations, the C-8-*R*-isomer (*R*-epimer), and the C-8-*S*-isomer (*S*-epimer). Toxic effects of ergot, such as vasoconstriction, have been primarily attributed to the *R*-epimer bioactivity, as compared to the *S*-epimer. Recent studies demonstrated potential bioactivity of *S*-epimers. Therefore, further cost-effective investigations of the *S*-epimers are needed. The present study investigated the *S*-epimer - vascular receptor binding relationship. An *in silico* molecular docking approach, utilizing AutoDock Vina and DockThor, was used to determine if the *S*-epimer (ergocristinine) binds to vascular receptors and to compare the binding affinity and interactions to the corresponding *R*-epimer (ergocristine) and a structural analogue (lysergic acid amide). The binding energy (kcal/mol) of ergocristinine was − 9.7 or − 11.0 to the serotonin (5-HT) 2 A receptor and − 8.7 or − 11.4 to the alpha 2 A adrenergic receptor, depending on the software used. A hydrogen bond was formed between ergocristinine and amino acid residues of the 5-HT 2 A and alpha 2 A adrenergic receptor binding sites, with bond lengths of 3.10 Å and 3.28 Å, respectively. Binding affinities and molecular interactions among the ligands to each receptor differed. Different affinities and interactions may relate to differences in the chemical structures. The binding affinities and strong molecular interactions of the *S*-epimer to vascular receptors may contribute to the observed physiological manifestations that occur after ergot alkaloid exposure. The results of the present study suggest further investigation on the receptor binding of the *S*-epimers of ergot alkaloids.

## Highlights

• An *in silico* approach was utilized to investigate the *S*-epimer, ergocristinine.
• Ergocristinine binds to vascular receptors with strong molecular interactions.
• This epimer – receptor interaction has not been demonstrated previously.
• The receptor interactions differed from ergocristine and lysergic acid amide.

## Graphical Abstract

**Figure:**
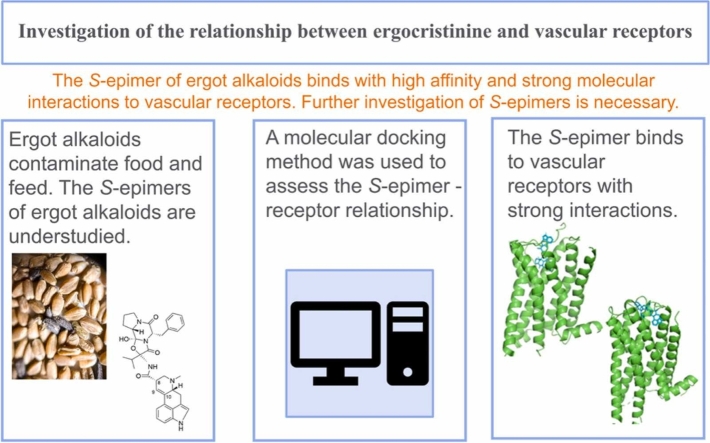

## 1 Introduction

Fungi, from the *Claviceps* genus, infect cereal crops or grasses [1], [2] intended for food or feed for humans or animals [3]. A fungal mass, known as ergot sclerotia, develop on the spike of the plant [2], [4]. Ergot alkaloids are toxic secondary metabolites that are produced by the fungus and located in the sclerotia. Multiple ergot alkaloids are produced by the fungi, differing in their chemical structure. Ergopeptines are a class of ergot alkaloids structurally consisting of an ergoline and amino acid ring system [5]. There are five different common ergopeptines produced by *Claviceps purpurea* that differ by substituents [6], [7]. Ergopeptine alkaloids exist in two configurations associated with rotation at the carbon 8 adjacent to the 9–10 carbon double bond of the alkaloid structure [8]. Each configuration is referred to as the C-8-(*R*)-isomer (*R*-epimer) or the C-8-(*S*)-isomer (*S*-epimer). Names of specific epimers have a different suffix, depending on the configuration. The *R*-epimers have a -ine suffix and the *S*-epimers have a -inine suffix.

The *R*-epimers of ergot alkaloids were deemed the bioactive configuration, whereas the *S*-epimers were deemed less or non-bioactive [5], [9], [10]. Studies referencing the non-bioactivity of *S*-epimers are dated [11], [12]. The rationale for the non-bioactivity of the *S*-epimers is unclear. Recent studies have suggested potential bioactivity of the *S*-epimers [13], [14], [15]. Previous *S*-epimers of non ergopeptine alkaloids have demonstrated biological activity [16], [17]. An ergoline ergot alkaloid, 8S-lisuride, demonstrated high affinity, and higher affinity than the corresponding epimer, to a histamine receptor [16]. Therefore, further investigation into the *S*-epimers of ergopeptines is warranted.

There is a large percentage of *S*-epimers, out of the total ergot alkaloid concentration, in ergot contaminated matrices [18], [19]. In addition, certain *R* and *S*-epimers of ergot alkaloids are more relevant than others. Ergocristine and ergocristinine are found at high concentrations within ergot sclerotia globally [6], [19]. Ergocristinine had the highest and second highest mean concentration in feed and wheat samples compared to all other *R* and *S*-epimers analyzed The *R*- and *S*-epimers can interconvert to one another [20], however, ergocristinine has demonstrated stability in terms of epimerization under physiological conditions [13]. Furthermore, ergocristine had the highest antagonist activity in an *ex vivo assay* [21]and was the most cytotoxic compared to other ergot alkaloids [22].

The *R*-epimers of ergot alkaloids have been associated with adverse health effects as a consequence of the consumption of ergot contaminated food and feed. Ergot alkaloids share a similar chemical structure to endogenous molecules; therefore, bind to receptors in humans and animals leading to adverse effects [5]. Serotonin (5-HT) and alpha-adrenergic receptors mediate vasoconstriction through ergot alkaloid binding [23], [24], [25]. The *R*-epimers are known to bind to receptors to produce a biological response. Ergot alkaloids have a high affinity for their target receptors [24], [26], [27], [28], [29], however, previous studies have only focused on *R*-epimers. Limited studies have assessed *S*-epimer receptor interactions *in vitro*, however, some studies assessing non ergopeptine ergot alkaloids have demonstrated agonist, and antagonist properties with relatively high affinity to vascular receptors [17], [30]. Since *in vitro* binding studies can be costly and resource consuming, *in silico* methods can be utilized.

Molecular docking, an *in silico* approach, assesses ligand-receptor interactions and the strength of the interactions [31]. Molecular docking software uses binding energy, which is termed binding affinity, for the scoring function [32], [33], [34], [35], [36]. The higher the negative number, the stronger the binding affinity of the ligand to a receptor [37]. The bound ligand to a receptor after molecular docking may be visualized to assess the molecular interactions between the ligand and the amino acid residues of the receptor binding site. Molecular docking is an inexpensive and time effective approach which could be useful in determining the relationship of expensive ergot alkaloids to various receptors, including both *R* and *S*-epimers.

Few studies have utilized a molecular docking approach to assess the interaction of ergot alkaloids to specific receptors. The binding of *R*-epimers and derivatives to receptors were investigated [28], [38], [39], however, the corresponding *S*-epimer binding was not assessed. One study assessed the affinity of ergotamine (*R*-epimer), and ergotaminine (*S*-epimer) to specific receptors [40]. The authors acknowledge there is a lack of *S*-epimer data concerning receptor binding. Another study screened multiple mycotoxins, including several *R* and *S*-epimers of ergot alkaloids, binding to nuclear receptors [41]. In each study, the *S*-epimer-receptor binding was not the primary focus. Therefore, the interaction of the *S*-epimers to receptors should be assessed in more detail.

**Fig. 1:**
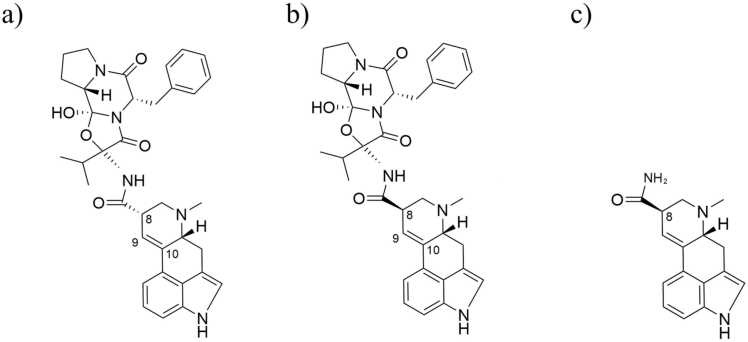
Structures of a) ergocristinine, b) ergocristine, and c) lysergic acid amide.

The aim of this study was to assess if ergocristinine (*S*-epimer) binds to the vascular receptors, 5-HT 2 A and alpha 2 A adrenergic, and to compare the binding affinity and molecular interactions of ergocristinine to ergocristine (*R*-epimer), and lysergic acid diamide (structural analogue), utilizing AutoDock Vina and DockThor molecular docking software. To the authors knowledge, molecular docking assessment of ergocristinine to vascular receptors has not been evaluated. Moreover, since the *S*-epimers of ergot alkaloids are deemed inactive, but recent studies are demonstrating potential activity of the *S*-epimers, further investigation into the potential binding relationship to important biological receptors should be evaluated. The *R*-epimers bind to receptors, producing a biological response. The *S*-epimers are costly, therefore, the results of an *in silico* method may advise if future receptor binding assays are needed. The structures of ergocristinine, ergocristine and lysergic acid amide are presented in Fig. 1. The experiments were validated through redocking and assessing the relationship of the predicted binding affinities to experimental binding affinities.

## 2 Materials and methods

### 2.1 Receptor protein

To investigate an ergocristinine – receptor binding relationship, the three-dimensional crystal structures of the receptors proteins were downloaded from Protein Data Bank (PDB) in a PDB format. Serotonin 2 A (5-HT 2 A) and alpha 2 A adrenergic were selected as the receptor proteins (PDB ID: 7WC6 and 6KUY, respectively). If the protein structures were in complex with a ligand or other molecules, the ligand or molecules were removed. The 5-HT 2 A and alpha 2 A adrenergic receptor were selected because of previous literature suggesting involvement of those receptors with ergot alkaloid exposure [23], [25], [42]. Exposure of *R*-epimers to vasculature *ex vivo* has demonstrated the involvement of the 5-HT 2 A and alpha 2 A adrenergic receptors in vascular contraction. These receptor classes are found in high amounts in the central and peripheral vasculature systems, respectively. Vascular contracture, because of ergot alkaloid exposure, can lead to detrimental health effects such as reduced blood flow to vital organs, and potentially loss of limbs. Since the 5-HT 2 A and alpha 2 A adrenergic receptors are involved with producing vascular constriction after *R*-epimer exposure, these two relevant receptor proteins were used to assess their potential involvement with *S*-epimer-receptor binding.

### 2.2 Ligands

The three-dimensional conformer molecular structure for the *S* and *R*-epimer, ergocristinine and ergocristine, and the structural analogue lysergic acid amide, were downloaded online from PubChem (PubChem CID: 31116 and 7067483, and 442072, respectively). The ligands were downloaded and saved as a SDS file.

### 2.3 Molecular docking

AutoDock MGL Tools (Molecular Graphics Laboratory, The Scripps Research Institute, Version 1.5.7), AutoDock Vina and PyMOL (PyMOL Molecular Graphics System, Version 2.0 Schrödinger, LLC) software were utilized. Molecular docking was preformed using the crystal structures of 5-HT 2 A and alpha 2 A adrenergic with ergocristinine (*S*), ergocristine (*R*), and lysergic acid amide.

In brief, AutoDockTools was used to prepare the protein (receptors) for docking. To prepare the protein, water molecules were removed, and polar hydrogen and charges (Kollman) were added. The protein was saved as a PDBQT file. To prepare the ligands, utilizing Pymol, the file was converted to a PDB format. Utilizing AutoDockTools, the PDB file was saved as a PDBQT file and selected for docking.

Once the protein and ligand are in PDBQT file format, a grid for the protein was prepared. The grid is the region of the receptor that the docking (binding) took place. The exploratory grid dimensions were estimated based on where the binding pocket is according to previous literature [39], [40], and where the agonists were bound to the receptors in the PDB files. The grid dimensions and parameters for 5-HT 2 A were center_x = −26.16, center_y = -− 15.96, center_z = 145.00; size_x = 30, size_y = 30, size_z = 30; energy_range = 4, exhaustiveness = 8. The grid dimensions and parameters for alpha 2 A adrenergic were center_x = 27.28, center_y = 1.96, center_z = 50.45; size_x = 30, size_y = 30, size_z = 30; energy_range = 4, exhaustiveness = 8. Docking was executed as flexible ligand docking with rigid receptors, which is a common approach [43]. The ligand bound to the receptor was visualized using Pymol.

DockThor [44] is a molecular docking software is freely available through the webserver. DockThor is a well-known molecular docking software [45]. The DockThor software utilized the same ligand and protein receptor files from the AutoDock Vina method, however, were converted back to PDB format. The standard search algorithm for molecular docking was utilized which included 24 runs. Soft docking was not executed. The grid dimensions were the same as the AutoDock Vina method for each receptor analyzed, however, a grid size of 20 for each dimension was required for the docking to occur.

### 2.4 Ligand-protein interactions

To assess the interactions between the bound ligand and the receptor, LigPlot+ was utilized. The ligand-receptor complex from AutoDock Vina was observed in the software LigPlot+ v.2.2.5. The amino acids residues of the binding site of the receptor interacting with the ligand were visualized. Hydrophobic interactions and hydrogen bonds were observed. Protein-Ligand Interaction Profiler (PLIP) was utilized to confirm data from LigPlot+ and to further analyze the data. The PLIP is available online [46], [47]. The aforementioned complexes were uploaded to the PLIP website as directed and analyzed.

### 2.5 Validation

Redocking was executed by extracting the ligand within the PDB crystal structure, for each receptor, 5-HT 2 A and alpha 2 A adrenergic (PDB ID: 7WC6 and 6KUY, respectively). The ligands were redocked to each respective receptor using the AutoDock Vina and DockThor molecular docking methods. The redocked ligand and crystal structure ligand were visually assessed for similarity in conformation for each binding pose. The most similar redocked and crystal conformation was used to calculate the root mean square deviation (RMSD) between structures. This method of validation through redocking has been executed recently [48], [49]. To calculate the RMSD, DockRMSD was utilized [50]. Both docked and crystalized ligand structures were uploaded as MOL 2 files and the RMSD was calculated without the use of hydrogens.

Comparing the AutoDock Vina binding affinities and experimental binding affinities were executed as follows. The aforementioned of AutoDock Vina docking method was executed on eight known ligands to the respective receptors. The experimental affinities of those ligands were investigated online and within the literature [51], [52], [53]. Ligand details can be found in the supplemental material. A correlation between the predicted and experimental binding affinities was assessed using SPSS 23 (IBM SPSS Statistics for Windows, version 23, IBM Corp., Armonk, NY). A one-sample Kolmogorov Smirnov test was executed to determine if data was normally distributed (P > 0.05). Since data was normally distributed, a Pearson correlation was conducted, and the correlation was defined as significant at P < 0.05.

## 3 Results

### 3.1 Molecular docking

**Fig. 2.**
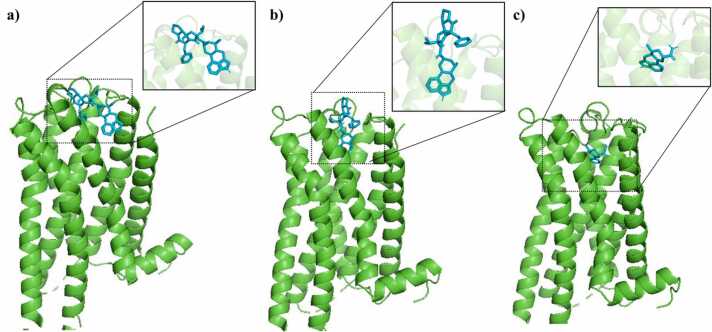
The binding of a) ergocristinine, b) ergocristine and c) lysergic acid amide to the serotonin (5-HT 2 A) receptor. The receptor protein is shown as a green colored ribbon cartoon, and the ligands are blue colored sticks. Side boxes highlight each ligand pose. (For interpretation of the references to color in this figure legend, the reader is referred to the web version of this article.)

**Fig. 3:**
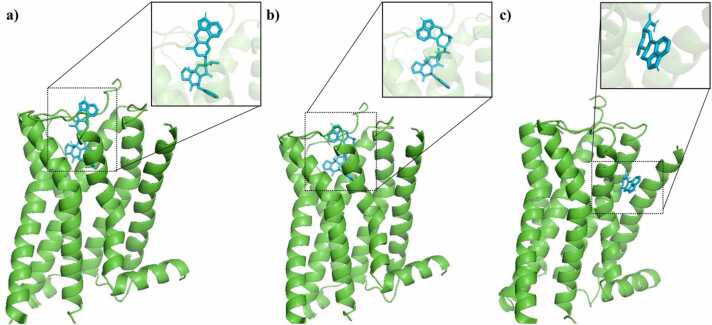
The binding of a) ergocristinine, b) ergocristine and c) lysergic acid amide to the alpha 2 A adrenergic receptor. The receptor protein is shown as a green colored ribbon cartoon, and the ligands are blue coloured sticks. Side boxes highlight each ligand pose. (For interpretation of the references to color in this figure legend, the reader is referred to the web version of this article.)

The AutoDock Vina predicted binding affinities for the 5-HT 2 A receptor were − 9.7 kcal/mol, − 10.2 kcal/mol and − 9.3 kcal/mol for ergocristinine, ergocristine, and lysergic acid amide, respectively (Fig. 2a,b,c). For the for the alpha 2 A adrenergic receptor, the binding affinities were − 8.7 kcal/mol, − 10.3 kcal/mol, and − 9.4 kcal/mol for ergocristinine, ergocristine and lysergic acid amide, respectively (Fig. 3a,b,c). A summary and the DockThor binding affinities for each ligand-receptor complex are presented in Table 1.

**Table 1:** The molecular docking binding affinities (kcal/mol) of ergocristinine, ergocristine and lysergic acid amide to the serotonin (5-HT) 2 A receptor and alpha 2 A adrenergic receptor using AutoDock Vina and DockThor software.

	5-HT 2 A		Alpha 2 A Adrenergic	
	AutoDock Vina	DockThor	AutoDock Vina	DockThor
Ergocristinine	-9.7	-11.0	-8.7	-11.4
Ergocristine	-10.2	-12.3	-10.3	-11.6
Lysergic Acid Amide	-9.3	-8.4	-9.4	-9.3

### 3.2 Ligand-receptor interactions

**Fig. 4:**
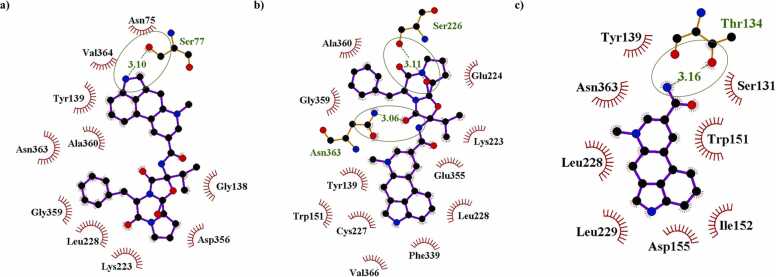
Molecular interactions of a) ergocristinine, b) ergocristine and c) lysergic acid amide to the 5-HT 2 A receptor. Ligands are represented in blue ball and stick. The amino acid residue of the receptor binding site with a hydrogen bond to the ligand is represented in green and circled. All other amino acid residues formed hydrophobic interactions. (For interpretation of the references to color in this figure legend, the reader is referred to the web version of this article.)

**Fig. 5:**
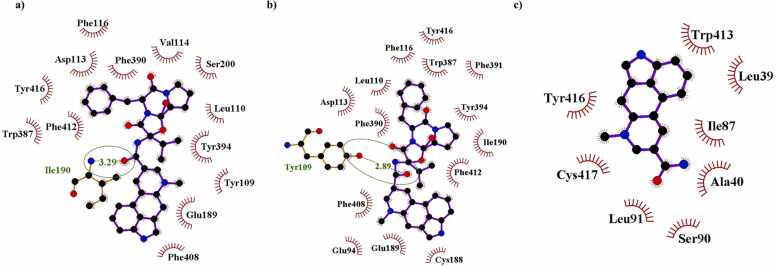
Molecular interactions of a) ergocristinine b) ergocristine and c) lysergic acid amide to the alpha 2 A adrenergic receptor. Ligands are represented in blue ball and stick. The amino acid residue of the receptor binding site with a hydrogen bond to the ligand is represented in green and circled. All other amino acid residues formed hydrophobic interactions. (For interpretation of the references to color in this figure legend, the reader is referred to the web version of this article.)

The amino acid residues of the 5-HT 2 A and alpha 2 A adrenergic receptors formed a greater number of hydrophobic interactions to ergocristinine and ergocristinine, compared to lysergic acid amide using LigPlot+ (Table 2). Amino acid residues, Ser77 and Ile190, formed a hydrogen bond with ergocristinine at the 5-HT 2 A and alpha 2 A adrenergic receptor binding sites, respectively. The hydrogen bond lengths were 3.10 Å (Figs. 4a) and 3.29 Å (Fig. 5a). Ergocristine had formed the greatest number of hydrogen bonds to the amino acid residues of the 5-HT 2 A receptor, compared to the other complexes (Fig. 4b). The Asn363 formed a hydrogen bond with a length of 3.06 Å and Ser226 formed a hydrogen bond with a length of 3.11 Å. Ergocristine had formed one hydrogen bond to the Tyr109 of the alpha 2 A adrenergic receptor with a length of 2.89 Å (Fig. 5b). One hydrogen bond was formed between lysergic acid amide and the 5-HT 2 A receptor (Fig. 4c), and no hydrogen bonds were formed to the amino acid residues of the alpha 2 A adrenergic receptor (Fig. 5c). A further summary of the molecular interactions can be found in the supplemental material (Supplementary Tables 1 and 2).

**Table 2:** The number of hydrophobic and hydrogen bond interactions between ergocristinine, ergocristine, and lysergic acid amide to the amino acid residues of the 5-HT 2 A and alpha 2 A adrenergic receptors using LigPlot+ .

	5-HT 2 A		Alpha 2 A Adrenergic	
	Hydrophobic Interactions	Hydrogen Bonds	Hydrophobic Interactions	Hydrogen Bonds
Ergocristinine	10	1	13	1
Ergocristine	11	2	14	1
Lysergic Acid Amide	8	1	8	0

Similar molecular interactions for each ligand receptor complex were observed using Protein-Ligand Interaction Profiler (PLIP) (Supplementary Tables 3 and 4). For the 5-HT 2 A and alpha 2 A adrenergic receptor, the same amino acid residues forming hydrogen bonds to ergocristine and ergocristinine as mentioned above, were observed. However, the bond distances were less according to the PLIP assessment. The Ser77 distance to ergocristinine was 2.07–2.1 Å and Ile190 was 2.67 Å for the 5-HT 2 A and alpha 2 A adrenergic receptors, respectively. The Asn363 and Ser226 distance to ergocristine had a range of 2.44–3.09 Å, for the 5-HT 2 A receptor. The hydrogen bond angles were closer to 180˚ for the 5-HT 2 A receptor, compared to the alpha 2 A adrenergic receptor. The PLIP tool further analyzes the protein-ligand complexes. Further hydrogen bonds, π-stacking, and a salt bridge between the amino acid residues of certain receptor-ligand complexes occurred.

### 3.3 Validation

The computational method of molecular docking has been validated and proven reliable [40]. The present study was successfully validated through redocking. Redocking was executed for each receptor, 5-HT 2 A and alpha 2 A adrenergic, using AutoDock Vina and DockThor software. AutoDock Vina and DockThor produced docking poses of the ligands that mimicked the ligands from the receptor crystalized structures. The RMSD between the visually similar docked ligand pose and the crystal structure ligand was < 2 Å for each receptor for both molecular docking software. A RMSD of < 2 Å between the docked and crystalized ligand is defined as acceptable and denotes pose similarity between ligands [48]. Therefore, the present study results are supported.

The comparison of AutoDock Vina predicted and experimental binding affinities using a large data set has previously been investigated and successfully demonstrated a relationship between the predicted and experimental binding affinities [26]. In the current study, a relationship between the AutoDock Vina binding affinities and experimental binding affinities was observed for each receptor. There was a significant correlation (P < 0.05) for the 5-HT 2 A receptor (Supplementary Fig. 1), and a nonsignificant (P > 0.05) correlation for the alpha 2 A adrenergic receptor (Supplementary Fig. 2). The correlation coefficients were 0.8 and 0.5 for the 5-HT 2 A and alpha 2 A adrenergic, respectively, demonstrating a strong to moderate correlation. The experimental binding affinities for the common ligands used for the 5-HT 2 A and alpha 2 A adrenergic receptor had varying affinity ranges (Supplementry Tables 5 and 6). The relationship between the docking scores and experimental affinities were based on the best possible outcome. If different experimental affinities were used, a relationship between the predicted binding affinities and the experimental affinities may not have occurred. Therefore, the variability of the experimental binding affinities indicates this method using a low number of ligands may be unreliable.

## 4 Discussion

Mycotoxins, such as ergot alkaloids, pose a threat to humans and animals through the consumption of ergot contaminated food and feed [2], [3]. Previously, the toxic effects of ergot alkaloids have been attributed to the *R*-epimers [5]. The *R*-epimers bind to vascular receptors causing vasoconstriction and subsequent adverse effects. Recently, studies have demonstrated vasoconstriction effects due to the *S*-epimers *ex vivo* [14], [15], warranting further *S*-epimer investigation. To the authors knowledge*, in vitro* studies assessing binding kinetics of the *S*-epimers of ergopeptine ergot alkaloids to vascular receptors has not been investigated, which may be due to the expensive nature of the *S*-epimers. The highlights of the present study are the use of an *in silico* method which demonstrated the *S*-epimer, ergocristinine, bound to two vascular receptors with strong interactions, and the receptor relationship differed from the corresponding *R*-epimer and structural analogue, which are discussed.

The use of an *in silico* method provides a cost and time effective approach to investigate a *S*-epimer – receptor relationship. A previous *in silico* study using ergotaminine (*S*-epimer) demonstrated binding affinities of − 7.8 kcal/mol and − 9.5 kcal/mol to the 5-HT 2 A and 5-HT 2B receptors using AutoDock Vina [40]. In the present study, ergocristinine (*S*-epimer) showed binding affinities of − 9.7 and − 8.7 kcal/mol to the 5-HT 2 A and alpha 2 A adrenergic receptors using AutoDock Vina. Differences in studies may be associated with the different *S*-epimers and receptors used. A binding energy value of − 7 represents a ligand having affinity for a receptor [41], and higher the negative value the stronger the binding affinity [37], which supports the results of the present study. In a different study utilizing ergocristinine, medium to high binding affinity to certain nuclear receptors was demonstrated [41], suggesting ergocristinine has the potential to have high affinity to receptors.

Short hydrogen bonds between a ligand and amino acid residues of a receptor binding site result in a strong interaction between the ligand and receptor [54]. The present study demonstrated shorter hydrogen bond distances between the *S*-epimer and the amino acid residues of both receptors compared to a previous study assessing ergotamine and 5-HT 1/2B receptors [38], [39]. The previous studies reported the hydrogen bonds provided stability to the epimer and strengthened the interactions, which supports the strong molecular interactions observed in the present study. The current study also showcased hydrogen bond angles close to 180˚, which results in strong interactions between the ligands and receptors [55]. The cost effective *in silico* analysis has demonstrated a significant *S-*epimer – vascular receptor relationship through assessing the binding affinity and strong molecular interactions, which has not been demonstrated previously.

In comparison to the *S*-epimers of ergot alkaloids, the *R*-epimers have been studied to a greater extent. The *R*-epimers of ergot alkaloids and derivatives have demonstrated high affinity to vascular receptors [29], [42], [56], [57], and higher affinity than endogenous ligands. The *R*-epimers have also demonstrated slow association and dissociation to vascular receptors [42], [58], [59], [60], which may be associated to the high receptor binding affinity. Endogenous ligands, such as serotonin, are unable to displace a *R*-epimer once it is bound to a receptor [56], supporting the strength of the bond between the ligand and receptor. The AutoDock Vina affinity values for ergocristine in the present study are comparable to a previous study utilizing AutoDock Vina [40], supporting the results in the present study. Ergocristine demonstrated the highest apparent binding affinities to the receptors and were different compared to ergocristinine and lysergic acid amide, which may be associated with differing chemical structures.

All ergot alkaloids share an ergoline ring in their chemical structure, however, have varying side chains. The ergoline ring has been reported to be the functional group that binds to receptors [61]. Ergocristinine and ergocristine differ in chemical structure with the configuration at the chiral carbon 8 [62], and lysergic acid amide does not have the amino acid ring system [5]. The structural differences may explain the differences in affinity and molecular interactions observed between compounds. Lower affinity of lysergic acid derivatives to receptors, compared to the *R*-epimers, have been suggested previously [29], and is supported in the present study. Structural similarities/differences of ergot alkaloids and their derivatives have an impact on the relationship to receptors [7], [58], [63] and small differences in ligand-receptor binding may influence downstream effects [7], [64]. Ergocristinine, ergocristine, and lysergic acid (structurally similar to lysergic acid amide) have all demonstrated varying downstream effects on vasculature [15], [29]. The epimer – receptor interactions lead to the adverse physical manifestations observed after ergot alkaloid exposure.

The high affinity of ergot alkaloids to vascular receptors may cause long lasting adverse effects. Cattle that were removed from an ergot contaminated pasture demonstrated signs of vasoconstriction many days after removal [23], supporting the high affinity of ergot epimers to vascular receptors. Recovery of ergot alkaloids in feces were low in a study of lambs fed ergot contaminated feed [65]. The authors attributed the low recovery to urinary excretion. However, the bioaccumulation of ergot alkaloids in vascular tissue [64], or the high affinity to vascular receptors [28], could be a potential explanation. Both *R* and *S*-epimers have demonstrated sustained vascular contraction of blood vessels, which supports the affinity of the epimers to vascular receptors observed in the present study [15], [29]. The implications of the affinity of ergot alkaloids to vascular receptors and sustained vasoconstriction could lead to chronic health conditions. Alpha-adrenergic receptors are abundant in the peripheral vasculature [66], which is where clinical manifestations of gangrene can take place. The affinity of the ergot epimers to the alpha 2 A adrenergic receptor may support the physiological alterations that occur. The present study supports the *S*-epimer contribution to vasoconstriction, which has been previously associated with the *R*-epimer.

## 5 Conclusions

Ergot alkaloids are found in high concentrations in food and feed commodities worldwide. The less studied *S*-epimer needed further investigation based on the potential bioactivity. The *S*-epimer, ergocristinine, demonstrated affinity and strong molecular interactions to vascular receptors, which may contribute to the adverse effects previously associated with the *R*-epimers. The studied ligands had different binding affinities and interactions to the vascular receptors which may be associated with structural differences. Since *S*-epimers such as ergocristinine are costly, the use of a low-cost method is economically favorable. The results of the present study demonstrated an interaction between the *S*-epimer and vascular receptors, therefore, future studies should include further docking assessments, molecular dynamics on the most promising complexes, and biological experiments to investigate and confirm the ergocristinine – receptor interactions. A limitation of the current study is the lack of protein conformation assessment which should be conducted in the future. The results of the present study have helped address the research gap of the *S*-epimers. Based on the high affinities and strong molecular interactions of the *S*-epimer to the vascular receptors, future assessments into the *S*-epimers of ergot alkaloids are encouraged.

## Funding

This research was supported by the 10.13039/501100000038Natural Sciences and Engineering Research Council of Canada (NSERC), Canada Graduate Scholarships - Doctoral (CGS D) Program and the Saskatchewan Ministry of Agriculture - Agriculture Development Fund (ADF) (grant number: 20180361).

## CRediT authorship contribution statement

**Jensen E. Cherewyk:** Data curation, Writing – original draft, Investigation, Validation, Formal analysis, Conceptualization, Methodology, Visualization. **Barry R. Blakley:** Supervision, Funding acquisition, Writing – review & editing, Resources. **Ahmad N. Al-Dissi:** Supervision, Funding acquisition, Writing – review & editing, Resources.

## Declaration of Competing Interest

The authors declare that they have no known competing financial interests or personal relationships that could have appeared to influence the work reported in this paper.

## Data Availability

Data will be made available on request.
