# Supplementary material for: Investigation of the relationship between ergocristinine and vascular receptors
Source: Toxicol Rep. 2023 May 14;10:604–11. doi: 10.1016/j.toxrep.2023.05.005 (PMC10199403; doi:10.1016/j.toxrep.2023.05.005)
Supplement: Supplementary file 1 — Supplementary material [file mmc1.docx]

**Supplemental Material**

Investigation of the relationship between ergocristinine and vascular receptors

Jensen E. Cherewyk^a,*^, Barry R. Blakley^a^ and Ahmad N. Al-Dissi^b^

^a^Department of Veterinary Biomedical Sciences, Western College of Veterinary Medicine, University of Saskatchewan, Saskatoon, SK, S7N 5B4, Canada; brb237@mail.usask.ca

^b^Department of Veterinary Pathology, Western College of Veterinary Medicine, University of Saskatchewan, Saskatoon, SK, S7N 5B4, Canada; ahmad.aldissi@usask.ca

^*^Correspondence: jensen.cherewyk@usask.ca

**Supplementary Table 1**

Summary of molecular interactions of ergocristinine, ergocristine, and lysergic acid amide to 5-HT (serotonin) 2A receptor using LigPlot+.

| **Ligand** | **Total molecular interactions** | **All interacting amino acid residues^1^** | **Hydrogen bonds** | **Amino acid residues with hydrogen bond** | **Hydrogen bond length** |
| --- | --- | --- | --- | --- | --- |
| Ergocristinine | 11 | *Asn363*,  *Ala360*,  *Tyr139*,  Val364,  Asn75,  Ser77,  Gly138,  Asp356  *Lys223*,  *Leu228*,  *Gly359* | 1 | Ser77 | 3.10 |
| Ergocristine | 13 | *Asn363*,  *Gly359*,  *Ala360*,  Ser226,  Glu224,  *Lys223*,  Glu355,  *Leu228*,  Phe339,  Val366,  Cys227,  *Trp151*,  *Tyr139* | 2 | Ser226, Asn363 | 3.11, 3.06 |
| Lysergic Acid Amide | 9 | *Leu228*,  *Asn363*,  *Tyr139*,  Thr134,  Ser131,  *Trp151*,  Ile152,  Asp155,  Leu229 | 1 | Thr134 | 3.16 |

^1^Italicized amino acid residues are common between at least two of the ligands.

**Supplementary Table 2**

Summary of molecular interactions of ergocristinine, ergocristine, and lysergic acid amide to alpha 2A adrenergic receptor using LigPlot+.

| **Ligand** | **Total molecular interactions** | **All interacting amino acid residues** | **Hydrogen bonds** | **Amino acid residues with hydrogen bond** | **Hydrogen bond distance** |
| --- | --- | --- | --- | --- | --- |
| Ergocristinine | 14 | Trp387, *Phe412*, *Tyr416*, *Asp113*, *Phe116*, *Phe390*, Val114, Ser200, Seu110, *Tyr394*, *Tyr109*, *Glu189*,  *Phe408*,  *Ile190* | 1 | Ile190 | 3.29 |
| Ergocristine | 15 | *Asp113*, *Tyr109*,  *Phe390*,  Leu110,  *Phe116*,  *Tyr416*,  Trp387,  Phe391,  *Tyr394*,  *Ile190*,  *Phe412*,  Cys188  *Glu189*  Glu94,  *Phe408* | 1 | Tyr109 | 2.89 |
| Lysergic Acid Amide | 9 | Tryp413,  Leu39,  Ile87,  Ala40,  Ser90,  Leu91,  Cys417, | 0 |  |  |

^1^Italicized amino acid residues are common between at least two of the ligands.

**Supplementary Table 3**

Molecular interactions of ergocristinine, ergocristine, and lysergic acid amid to 5-HT (serotonin) 2A receptor using Protein-Ligand Interaction Profiler (PLIP).

| **Ergocristinine** | | |  | **Ergocristine** | | |  | **Lysergic Acid Amide** | | |  |
| --- | --- | --- | --- | --- | --- | --- | --- | --- | --- | --- | --- |
| **HP^1^** |  |  |  | **HP** |  |  |  | **HP** |  |  |  |
| Residue | AA^2^ | Distance (Å) |  | Residue | AA | Distance (Å) |  | Residue | AA | Distance (Å) |  |
| 139 | TYR | 3.78 |  | 223 | LYS | 3.66 |  | 151 | TRP | 3.38 |  |
| 223 | LYS | 3.68 |  | 224 | GLU | 3.58 |  | 152 | ILE | 3.57 |  |
| 228 | LEU | 3.7 |  | 228 | LEU | 3.61 |  | 152 | ILE | 3.87 |  |
| 228 | LEU | 3.74 |  | 339 | PHE | 3.74 |  | 155 | ASP | 3.84 |  |
| 360 | ALA | 3.99 |  | 339 | PHE | 3.26 |  |  |  |  |  |
| 364 | VAL | 3.72 |  | 360 | ALA | 3.79 |  |  |  |  |  |
| **H^3^** |  |  |  | **H** |  |  |  | **H** |  |  |  |
| Residue | AA | Distance H-A^4^,  D-A^5^ (Å) | A  (˚)^6^ | Residue | AA | Distance H-A,  D-A (Å) | A  (˚) | Residue | AA | Distance H-A,  D-A (Å) | A  (˚) |
| 77 | SER | 2.17,3.1 | 169.18 | 226 | SER | 3.09,3.97 | 154.62 | 229 | LEU | 2.95,3.89 | 153.52 |
| 77 | SER | 2.1,3.1 | 166.17 | 226 | SER | 2.79,3.79 | 170.06 |  |  |  |  |
| 363 | ASN | 3.19,4.05 | 143.03 | 363 | ASN | 2.24,3.06 | 136.34 |  |  |  |  |
|  |  |  |  | 363 | ASN | 2.44,3.34 | 154.63 |  |  |  |  |
| **π-S^7^** |  |  |  | **π-S** |  |  |  |  |  |  |  |
| Residue | AA | Distance (Å) |  | Residue | AA | Distance (Å) |  |  |  |  |  |
| 139 | TYR | 5.15 |  | 139 | TYR | 4.92 |  |  |  |  |  |

^1^Hydrophobic interactions. ^2^Amino acid type. ^3^Hydrogen bonds. ^4^Hydrogen and acceptor atom distance. ^5^Donor and acceptor atom distance. ^6^Angle between donor, acceptor and hydrogen atoms. ^7^ π-Stacking.

**Supplementary Table 4**

Molecular interactions of ergocristinine, ergocristine, and lysergic acid amid to alpha adrenergic 2A receptor using Protein-Ligand Interaction Profiler (PLIP).

| **Ergocristinine** | | |  | **Ergocristine** | | |  | **Lysergic Acid Amide** | | |  |
| --- | --- | --- | --- | --- | --- | --- | --- | --- | --- | --- | --- |
| **HP^1^** |  |  |  | **HP** |  |  |  | **HP** |  |  |  |
| Residue | AA^2^ | Distance (Å) |  | Residue | AA | Distance (Å) |  | Residue | AA | Distance (Å) |  |
| 109A | TYR | 3.35 |  | 109A | TYR | 3.97 |  | 39A | LEU | 3.58 |  |
| 110A | LEU | 3.71 |  | 110A | LEU | 3.44 |  | 40A | ALA | 3.76 |  |
| 114A | VAL | 3.77 |  | 113A | ASP | 3.79 |  | 413A | TRP | 3.52 |  |
| 116A | PHE | 3.54 |  | 116A | PHE | 3.52 |  | 413A | TRP | 3.79 |  |
| 189A | GLU | 3.28 |  | 189A | GLU | 3.75 |  | 413A | TRP | 3.68 |  |
| 387A | TRP | 3.78 |  | 190A | ILE | 3.49 |  | 413A | TRP | 3.68 |  |
| 390A | PHE | 3.22 |  | 190A | ILE | 3.32 |  |  |  |  |  |
| 408A | PHE | 3.76 |  | 387A | TRP | 3.44 |  |  |  |  |  |
| 416A | TYR | 3.16 |  | 387A | TRP | 3.26 |  |  |  |  |  |
|  |  |  |  | 408A | PHE | 3.24 |  |  |  |  |  |
|  |  |  |  | 408A | PHE | 3.95 |  |  |  |  |  |
|  |  |  |  | 412A | PHE | 3.4 |  |  |  |  |  |
|  |  |  |  | 412A | PHE | 3.66 |  |  |  |  |  |
|  |  |  |  | 416A | TYR | 2.97 |  |  |  |  |  |
| **H^3^** |  |  |  | **H** |  |  |  | **H** |  |  |  |
| Residue | AA | Distance H-A^4^,  D-A^5^ (Å) | A  (˚)^6^ | Residue | AA | Distance H-A,  D-A (Å) | A  (˚) | Residue | AA | Distance H-A,  D-A (Å) | A (˚) |
| 190A | ILE | 2.67,3.29 | 118.87 | 109A | TYR | 2.15,2.89 | 133.99 | 40A | ALA | 3.25,3.96 | 127.36 |
|  |  |  |  | 109A | TYR | 2.55,2.89 | 100.54 |  |  |  |  |
| **π-S^7^** |  |  |  | **π-S** |  |  |  |  |  |  |  |
| Residue | AA | Distance (Å) |  | Residue | AA | Distance (Å) |  |  |  |  |  |
| 387A | TRP | 4.81 |  | 390A | PHE | 4.61 |  |  |  |  |  |
| 390A | PHE | 4.87 |  |  |  |  |  |  |  |  |  |
|  |  |  |  | **Salt Bridge** |  |  |  |  |  |  |  |
|  |  |  |  | Residue | AA | Distance (Å) |  |  |  |  |  |
|  |  |  |  | 94A | GLU | 5.48 |  |  |  |  |  |

^1^Hydrophobic interactions. ^2^Amino acid type. ^3^Hydrogen bonds. ^4^Hydrogen and acceptor atom distance. ^5^Donor and acceptor atom distance. ^6^Angle between donor, acceptor and hydrogen atoms. ^7^ π-Stacking.

**Supplementary Table 5**

Predicted binding affinities of common ligands to the 5-HT (serotonin) 2A receptor utilizing AutoDock Vina and experimental binding affinities.

| **Ligand** | **1st Docking Score (kcal/mol)** | **2nd Docking Score (kcal/mol)** | **Experimental Binding Affinity (pK_i_)^1^** | **Species Used in Experiment** | **Ligand PubChem ID** |
| --- | --- | --- | --- | --- | --- |
| LSD | -9.7 | -9.5 | 9.06^2^ | n/a | 5761 |
|  |  |  | 9.4 | human |  |
|  |  |  | 8.5 | rat |  |
| Ergotamine | -10.7 | -10.4 | 9 | human | 8223 |
|  |  |  | 8 | rat |  |
| 5-HT | -6.6 | -6.6 | 6.0-8.4 | human | 5202 |
|  |  |  | 6.0-6.5 | Rat |  |
|  |  |  |  |  |  |
| Bromocriptine | -10.2 | -8.8 | 7 | human | 31101 |
| Lorcaserin | -7.4 | -6.9 | 6.8 | human | 11658860 |
| methylergonovine | -9.6 | -9.5 | 9.4 | human | 8226 |
|  |  |  | 7.9 | rat |  |
| DOI | -6.1 | -6 | 7.4-9.2 | human | 170617 |
|  |  |  | 7.1 | rat |  |
| Terguride | -9.7 | -9.2 | 8.3 | human | 443951 |

^1^IUPHAR/BPS Guide to Pharmacology (<https://www.guidetopharmacology.org/GRAC/ObjectDisplayForward?objectId=6>). ^2^Paulke et al., 2013.


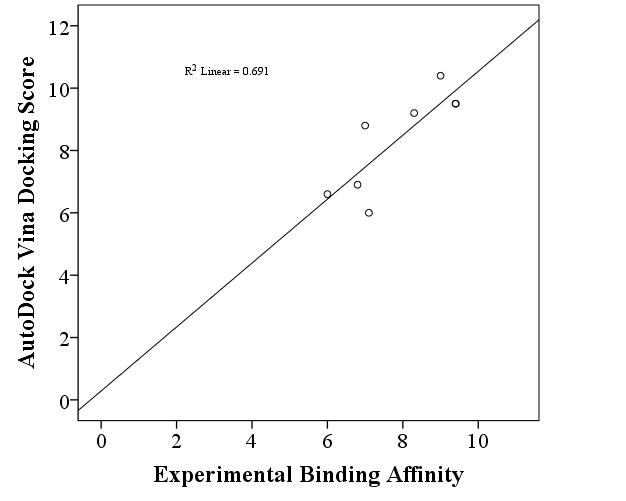


**Supplementary Fig. 1.** Predicted AutoDock Vina docking score (binding affinity) and experimental binding affinity for ligands to the 5-HT 2A receptor. AutoDock Vina Scores were taken as absolute values for ease of interpretation. If there was a range for the experimental binding affinity for a ligand, the value taken was the most similar value to the AutoDock Vina scores to investigate a potential relationship. Data was normality distributed (One-Sample Kolmogorov-Smirnov Test, P > 0.05) and was significantly correlated (Pearson Correlation = 0.831, P < 0.05, n = 8, SPSS version 23).

**Supplementary Table 6**

Predicted binding affinities of common ligands to the alpha 2A adrenergic receptor utilizing AutoDock Vina and experimental binding affinities.

| **Ligand** | **1st Docking Score (kcal/mol)** | **2nd Docking Score (kcal/mol)** | **Experimental Binding Affinity (pK_i_)^1^** | **Species Used in Experiment** | **Ligand PubChem ID** |
| --- | --- | --- | --- | --- | --- |
| LSD | 9.2 | 9.5 | 8.99^2^ | n/a | 5761 |
| apraclonidine | 6.7 | 6.7 | 8.5 | human | 2216 |
| Iofexidine | 6.9 | 6.9 | 8.4 | human | 30668 |
| pergolide | 9 | 8.2 | 7.3 | human | 47811 |
| adrenaline | 6.3 | 6.3 | 5.8-7.4 | human | 5816 |
| Guanfacine | 7.4 | 7.4 | 7.1-7.3 | human | 3519 |
| Clonidine | 6.7 | 6.7 | 7.2-9.2 | human | 2803 |
| Dexmedetomidine | 7.5 | 7.4 | 7.6-9.6 | human | 5311068 |

^1^IUPHAR/BPS Guide to Pharmacology (<https://www.guidetopharmacology.org/GRAC/ObjectDisplayForward?objectId=25>). ^2^Paulke et al., 2013.


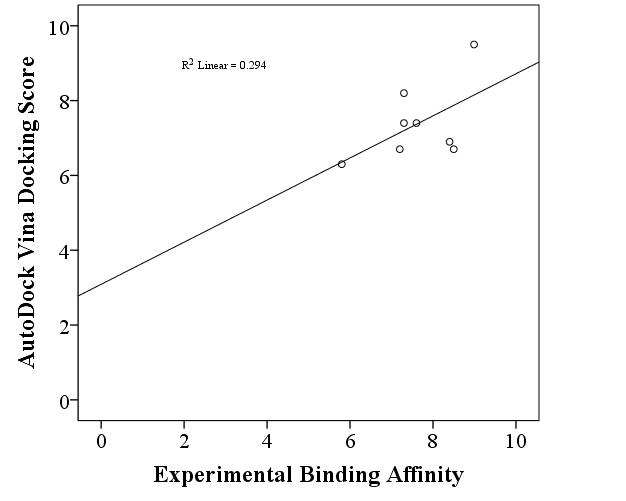


**Supplementary Fig. 2.** Predicted AutoDock Vina docking score (binding affinity) and experimental binding affinity for ligands to the alpha 2A adrenergic receptor. AutoDock Vina Scores were taken as absolute values for ease of interpretation. If there was a range for the experimental binding affinity for a ligand, the value taken was the most similar value to the AutoDock Vina scores to investigate a potential relationship. Data was normality distributed (One-Sample Kolmogorov-Smirnov Test, P > 0.05) and was not significantly correlated (Pearson Correlation = 0.542, P > 0.05, n = 8, SPSS version 23).

**References**

A. Paulke, C. Kremer, C. Wunder, J. Achenbach, B. Djahanschiri, A. Elias, J.S. Schwed, H. Hübner, P. Gmeiner, E. Proschak, S.W. Toennes, H. Stark, *Argyreia nervosa* (Burm. f.): Receptor profiling of lysergic acid amide and other potential psychedelic LSD-like compounds by computational and binding assay approaches, J. Ethnopharmacol. 148 (2013) 492–497. <https://doi.org/10.1016/j.jep.2013.04.044>.

K. Altosaar, P. Balaji, R.A. Bond, D.B. Bylund, S. Cotecchia, D. Devost, V.A. Doze, D.C. Eikenburg, S. Gora, E. Goupil, R.M. Graham, T. Hébert, J.P. Hieble, R. Hills, S. Kan, G. Machkalyan, M.C. Michel, K.P. Minneman, S. Parra, D. Perez, R. Sleno, R. Summers, P. Zylbergold. Adrenoceptors (version 2019.3) in the IUPHAR/BPS Guide to Pharmacology Database. IUPHAR/BPS Guide to Pharmacology. 3 (2019). Available online : <https://doi.org/10.2218/gtopdb/F4/2021.3> (accessed on: 07-01-23).

R. Andrade, N.M. Barnes, G. Baxter, J. Bockaert, T. Branchek, A. Butler, M.L. Cohen, A. Dumuis, R.M. Eglen, M. Göthert, M. Hamblin, M. Hamon, P.R. Hartig, R. Hen, J. Hensler, K. Herrick-Davis, R. Hills, D. Hoyer, P.P.A. Humphrey, K.P. Latté, L. Maroteaux, G.R. Martin, D.N. Middlemiss, E. Mylecharane, J. Neumaier, S.J. Peroutka, J.A. Peters, B. Roth, P.R. Saxena, T. Sharp, A. Sleight, C.M. Villalon, F. Yocca. 5-Hydroxytryptamine receptors (version 2019.4) in the IUPHAR/BPS Guide to Pharmacology Database. IUPHAR/BPS Guide to Pharmacology. 4 (2019). Available online: https://doi.org/10.2218/gtopdb/F1/2019.4(accessed on: 07-01-23).
